# Current Screening Strategies for Pancreatic Cancer

**DOI:** 10.3390/biomedicines10092056

**Published:** 2022-08-23

**Authors:** Petr Vanek, Ondrej Urban, Vincent Zoundjiekpon, Premysl Falt

**Affiliations:** 2nd Department of Internal Medicine—Gastroenterology and Geriatrics, Faculty of Medicine and Dentistry, Palacky University and University Hospital, Olomouc, I.P. Pavlova 185/6, 77900 Olomouc, Czech Republic

**Keywords:** pancreas, pancreatic cancer, pancreatic ductal adenocarcinoma, hereditary pancreatic cancer, screening, diagnosis

## Abstract

Pancreatic ductal adenocarcinoma (PDAC) is a dreaded malignancy with a dismal 5-year survival rate despite maximal efforts on optimizing treatment strategies. Radical surgery is the only potential curative procedure. Unfortunately, the majority of patients are diagnosed with locally advanced or metastatic disease, which renders them ineligible for curative resection. Early detection of PDAC is thus considered to be the most effective way to improve survival. In this regard, pancreatic screening has been proposed to improve results by detecting asymptomatic stages of PDAC and its precursors. There is now evidence of benefits of systematic surveillance in high-risk individuals, and the current guidelines emphasize the potential of screening to affect overall survival in individuals with genetic susceptibility syndromes or familial occurrence of PDAC. Here we aim to summarize the current knowledge about screening strategies for PDAC, including the latest epidemiological data, risk factors, associated hereditary syndromes, available screening modalities, benefits, limitations, as well as management implications.

## 1. Introduction

**Figure 1:**
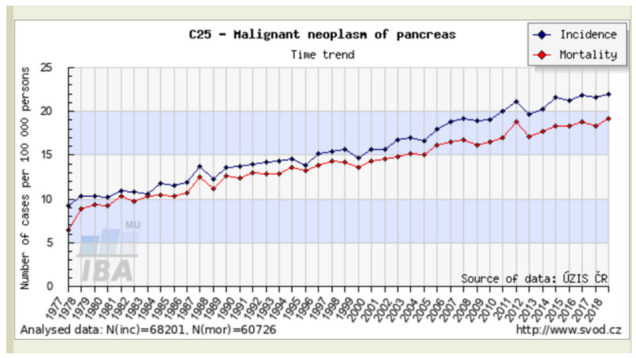
Incidence and mortality trends of malignant pancreatic neoplasms in the Czech Republic [3].

Pancreatic ductal adenocarcinoma (PDAC) represents the majority of malignant pancreatic neoplasms and has one of the worst prognoses among solid malignancies. Based on the GLOBOCAN 2020 estimates, it is the seventh leading cause of cancer-related death in both men and women worldwide with 496,000 new cases and an almost identical mortality rate when accounting for 466,000 deaths [1]. Many countries have been witnessing a steady increase in both incidence and mortality, likely reflecting the rising prevalence of obesity, diabetes, and alcohol consumption, albeit the advancement in diagnosis along with available cancer registries may also be a factor [1,2]. Reported rates are four- to five-fold higher in regions with high socio-demographic indices, with the highest incidence in Europe, North America, Australia, and New Zealand [1]. The time trend of malignant pancreatic neoplasms in the Czech Republic is demonstrated in Figure 1 [3]; in 2018 it was the seventh most frequently diagnosed malignancy with 2332 new cases and the third most common cause of cancer mortality with 2159 deaths, which ranked third in Europe [4]. In the United States, PDAC is currently the third leading cause of cancer death after lung cancer and colorectal cancer, and it is predicted to be the second deadliest cancer by 2030 [5,6].

In current practice, the diagnosis of PDAC is frequently delayed, as symptoms are often few, if any, and vague. Consistent with this fact, the majority of PDAC patients are diagnosed late with poor prognosis, as most patients (85–90%) present with either locally advanced (unresectable) or metastatic disease at detection [7,8]. The 5-year survival rate in the case of metastatic, regional, and localized disease is 3%, 14.4%, and 41.6%, respectively [9]. The dreary prognosis also reflects its aggressive tumor biology with low responsiveness to chemotherapy and radiation therapy [10,11,12]. Compared to other malignancies, scant improvements in the survival rate have been achieved in PDAC patients over the last decades, and radical surgical resection of localized disease remains the only curative approach [13,14,15].

Screening approaches with detection of asymptomatic stages of PDAC and its precursors have been proposed to improve the results. Current guidelines recommend against unselected screening for PDAC in the general population, concluding that potential benefits do not outweigh potential harms, as the incidence in persons at average risk is still relatively low and simple cost-effective screening tools are lacking [16,17,18]. On the other hand, individuals with an increased risk of PDAC based on family history or an identifiable genetic predisposition are clear targets for selective screening, and it has been recommended by major expert societies [15,16,17,18,19,20,21,22,23].

The purpose of this article is to provide an overview of the recommended screening approaches for PDAC, reviewing current epidemiological data, predisposing factors, associated genetic syndromes, available screening modalities, goals of screening, its benefits as well as limitations. Additionally, management implications including indications for surgical therapy are outlined.

## 2. Risk Factors

The lifetime risk of developing PDAC among the general population is approximately 1.5% [9]. A lifetime risk of >5% or relative risk (RR) > 5 have been accepted as the threshold to define high-risk individuals (HRI) for developing PDAC [23]. This threshold has been also widely acknowledged by guidelines and clinical practice updates to determine when PDAC screening is recommended [15,17,21,23].

Most cases of PDAC are sporadic, but 10–15% are estimated to be attributable to inherited risk factors [17,24,25]. The diagnosis of PDAC tends to aggregate in some families, and approximately 5–10% of individuals with PDAC have a positive family history [24,25]. Regarding the increased hereditary risk of PDAC, there are two main categories. The first includes rare defined syndromes of inherited cancer susceptibility, which account for about 20% of hereditary forms of PDAC. These are Peutz–Jeghers syndrome (PJS), familial atypical multiple mole and melanoma (FAMMM) syndrome, hereditary breast and ovarian cancer (HBOC) syndrome, Lynch syndrome, ataxia telangiectasia, and hereditary pancreatitis. The second category is familial pancreatic cancer (FPC), which accounts for the remaining 80%. In the case of genetic susceptibility syndromes, the degree of PDAC risk varies depending on the type of mutation; in FPC kindreds, the risk of developing the disease increases with the number of affected relatives [24].

Environmental risk factors for PDAC include dietary habits, obesity, type 2 diabetes mellitus (DM), excess alcohol consumption, chronic pancreatitis (CP), and tobacco use. Among lifestyle risk factors, cigarette smoking is considered the best established and most important preventable cause of PDAC [26,27]. In the case of sporadic PDAC, smoking increases the risk two- to three-fold, and it has been assumed that up to 25% of PDACs are associated with tobacco use [26,27]. In those with positive family history or genetic predispositions, smoking inflicts a greater effect (3.7-fold increased risk) and has been associated with an earlier diagnosis by up to 20 years [28,29]. Furthermore, cigarette smoking is an independent risk factor for the development of CP, where it is thought to accelerate disease progression by inducing chronic inflammation. The risk of PDAC in individuals with sporadic CP after 10 and 20 years of disease duration amounts to 1.8% and 4%, respectively [30,31]. An approximately two-fold increase in the risk of PDAC has been estimated for patients with a history of DM [32,33]. However, risk assessment in this regard is difficult given that DM can also be a paraneoplastic symptom. The risk of developing PDAC increases further with age; the median is 65 years [4].

## 3. Genetic Susceptibility Syndromes

### 3.1. Peutz–Jeghers Syndrome

Germline mutations in the *STK11* (*LKB1*) gene are associated with PJS, an autosomal dominant disease that results in numerous hamartomatous polyps throughout the gastrointestinal tract (GIT), orofacial melanin pigmentation, and various GIT malignancies. Patients with PJS have a 11–36% lifelong risk of developing PDAC, RR = 132 [34,35,36,37].

### 3.2. Familial Atypical Multiple Mole and Melanoma Syndrome

Germline mutations in the *CDKN2A* gene characterize FAMMM syndrome. This autosomal dominant genodermatosis is associated with numerous dysplastic nevi and malignant melanomas. The FAMMM variant increases the cumulative risk of PDAC to 17%, RR = 13–39 [37,38,39].

### 3.3. Hereditary Breast and Ovarian Cancer Syndrome

Germline mutations in the *BRCA1* and *BRCA2* genes indicate HBOC syndrome. They are inherited autosomal dominantly with high penetration. Compared to the general population, the risk of PDAC in the carriers of *BRCA1* germline mutations was reported to be three-fold, in *BRCA2* it was associated with RR of 3–9 [37]. Mutations in the *PALB2* gene, a partner gene of *BRCA2*, also increase the risk of PDAC. In a study by Yang and colleagues, this type of mutation was associated with a 2–3% risk of PDAC [40].

### 3.4. Lynch Syndrome

Germline mutations in mismatch repair genes, especially *MLH1*, *MSH2*, and *MSH6*, are associated with Lynch syndrome, a disease with an autosomal dominant type of inheritance with high penetration. Affected individuals develop early colorectal and endometrial cancers, but they are also at risk for other cancers, including PDAC. Kastrinos and colleagues demonstrated a 3.7% cumulative risk of PDAC in patients with Lynch syndrome; in another study, DaVee and colleagues reported an RR of 9–11 [37,41].

### 3.5. Ataxia Telangiectasia

Ataxia telangiectasia is a complex autosomal recessive syndrome caused by germline mutations in the *ATM* gene, which increase sensitivity of cells to potentially mutagenic environmental factors (e.g., sunlight) and susceptibility to malignant transformation. The disease is known for neurological manifestations and vascular anomalies, but the *ATM* variant carriers also have a 6.5-fold risk of PDAC [42].

### 3.6. Hereditary Pancreatitis

The term “hereditary pancreatitis” is typically used for an autosomal dominant disorder associated with germline mutations in the *PRSS1* gene. In a broader sense, hereditary pancreatitis can be also caused by mutations in other genes (e.g., *SPINK1*), which are associated with autosomal recessive inheritance. The disease represents a small proportion of CP cases, which in most people develop before the age of twenty and often before the age of five. Due to chronic inflammation, hereditary pancreatitis is associated with a markedly increased risk for PDAC [43]. The lifetime risk of PDAC for autosomal dominant variants is reported to be 25–44%, RR = 50–82 [44,45]. The risk in *SPINK1* and other mutations associated with hereditary pancreatitis is less well studied, but Muller and colleagues in their study including individuals with the *SPINK1* pathogenic variant found a 12-fold increase in PDAC risk [46].

## 4. Familial Pancreatic Cancer

FPC is an inherited predisposition to PDAC characterized by the accumulation of the disease in families. To meet the definition of FPC, there must be at least one pair of first-degree relatives (FDR) affected with PDAC, i.e., parent–child or siblings, in whom a defined genetic susceptibility syndrome has not been identified. Klein and colleagues prospectively analyzed data from an extensive FPC registry to determine the risk of PDAC in at-risk relatives [24]. Based on the number of affected relatives, they estimated RR to 4.6, 6.4, and 32 when one, two, and three FDRs were affected, respectively. Even though the causative gene variations in FPC have not been identified, previous modeling studies indicated an infrequent allele with autosomal dominant inheritance as possible etiology [47].

Individuals with positive history but not qualifying as FPC may also be at an increased risk for PDAC; an overall RR of 1.8 (95% CI, 1.48–2.12) was estimated for these subjects in a meta-analysis involving 6568 PDAC patients [48]. Nonetheless, pancreatic screening is not recommended for such individuals, as they may not benefit from screening [21].

## 5. Genetic Evaluation

Essentially all individuals should be assessed for the risk of an inherited predisposition to cancer. This evaluation includes detailed personal and family history with types of cancers in blood relatives and their age at diagnosis. In case there is a suspicion for the presence of hereditary cancer or the risk of its development based on anamnestic data, selected individuals should be referred for genetic counseling and germline testing as appropriate. In a recent provisional clinical opinion, the American Society of Clinical Oncology recommended targeted identification and surveillance of family members with a possible hereditary predisposition to PDAC [49]. It also included recommendations for universal genetic testing in all patients with PDAC regardless of family history. These recommendations were subsequently adopted by the National Comprehensive Cancer Network [50]. Pathogenic germline mutations in susceptibility genes are detected in approximately 4–20% of PDAC patients, including patients with clinically sporadic tumors without a positive family history [17,51,52,53,54,55,56,57]. The identification of hereditary cancer syndromes may not only affect indications for follow-up of the patient’s relatives, but it may change the patient care, as some mutations are potentially targetable with therapy, e.g., tumor responsiveness to poly (ADP-ribose) polymerase inhibitors in *BRCA1/2* gene abnormalities [58].

## 6. Pancreatic Cancer Screening in High-Risk Individuals

The decision to commence with pancreatic screening in individuals at an increased risk for PDAC requires discussion of the benefits, potential risks, and a relative paucity of definitive data on long-term outcomes. It is to be performed in an academic setting by an experienced multidisciplinary team and only for individuals who are candidates for surgery [15,17]. Starting age for screening varies based on the underlying genetic condition. Screening recommendations made by expert societies for individuals with an inherited risk of PDAC, including selected genetic susceptibility syndromes and family history criteria, are outlined in Table 1 [17,19,21]. Of note, the American Society for Gastrointestinal Endoscopy (ASGE) recently recommended not to require family history of PDAC in individuals with *BRCA1/2* pathogenic variants to be considered for pancreatic cancer screening, given that almost two in three *BRCA1/2*-positive individuals with PDAC do not have a positive family history and would have been missed [21]. The International Cancer of the Pancreas Screening (CAPS) Consortium failed to reach consensus on family history criteria for *BRCA1* mutation carriers but recommended that these carriers undergo surveillance; for carriers of mutations in *BRCA2* and *PALB2*, the consensus was to recommend surveillance for individuals who have a blood relative with PDAC [17].

### 6.1. Recommended Screening Modalities

The current PDAC screening strategy is based on imaging methods. A combination of endoscopic ultrasonography (EUS) and magnetic resonance imaging with magnetic resonance cholangiopancreatography (MR/MRCP) performed annually is recommended; computed tomography (CT) with the pancreatic protocol is indicated in individuals who cannot undergo EUS or MR/MRCP, mainly due to lower detection rates of smaller lesions and efforts to avoid ionizing radiation [15,17,21,23,59]. Some suggest alternating between EUS and MR/MRCP or choosing the modality based on patient preference and available expertise [21,23].

The U.S. Preventive Services Task Force performed a systematic analysis of screening studies [20]. EUS-based screening in nine studies had a diagnostic yield ranging from 0 (97.5% CI, 0.0–16.9) to 68.2 (95% CI, 14.3–186.6) cases per 1000 subjects [25,59,60,61,62,63,64,65,66]. MR/MRCP-based screening results were reported in eight studies and had a diagnostic yield from 0 (97.5% CI, 0.0–16.9) to 75 (95% CI, 15.7–203.9) cases per 1000 subjects [25,59,64,65,67,68,69]. Two studies evaluated CT, where the diagnostic yield was 0 (97.5% CI, 0.0–16.9) to 12.8 (95% CI, 0.3–69.4) cases per 1000 subjects [59,63]. In addition, EUS and MR/MRCP have proven to be complementary. MR/MRCP is particularly sensitive for the detection of cystic lesions and EUS for the detection of solid lesions with the possibility of tissue acquisition [65,70]. In a study by Canto and colleagues, however, only EUS detected stage I PDAC [71]. An advantage of EUS over CT may be the fact that some PDACs appear isodense on CT but are in fact identifiable on EUS [72]. Importantly, EUS has a very high negative predictive value [73,74]. This is valuable for clinicians indicating that EUS can also exclude PDAC, although it is an expert-dependent method. On the other hand, CT can quantify changes in visceral fat and lumbar muscles that may accompany the early stages of PDAC [75,76]; however, this approach has not been validated in prospective studies.

Endoscopic retrograde cholangiopancreatography (ERCP) is not recommended for screening due to the risk of post-ERCP pancreatitis (PEP) [17]. However, studies examining the benefits of ERCP are contradictory. In a prospective study by Canto and colleagues, performing ERCP in abnormal EUS findings provided no further clinically relevant information, and it was associated with a 7% rate of PEP [63]. In contrast, collecting pancreatic juice for cytology in the case of main pancreatic duct (MPD) caliber changes or small cystic lesions was beneficial in detecting early stages of PDAC among Japanese studies; the sensitivity, specificity, and accuracy of preoperative cytology were 75%, 100% and 88%, respectively [77,78]. Pancreatic juice, or cystic fluid, aspirated during EUS examination may be further analyzed by genetic sequencing. Potential markers include mutant GNAS, mutant KRAS, and mutant TP53 [79,80,81]. In a study involving HRIs and control subjects, Kanda and colleagues proved the presence of mutant TP53 in pancreatic juice in 29 of 43 PDAC patients, but in none among the controls [81].

### 6.2. Blood-Based Biomarkers

At present, there are no conclusive data to recommend a specific biomarker as a screening tool for PDAC. The only routinely used serological marker for the diagnosis of PDAC is carbohydrate antigen (CA) 19-9. Nevertheless, the sensitivity and specificity of CA 19-9 in the diagnosis of early PDAC is not high, which limits its clinical application. The marker maintains a sensitivity of 79–81% and a specificity of 82–90% for the diagnosis of PDAC in symptomatic patients [82,83], and its elevation signifies advanced disease and poor prognosis [84,85,86]. However, as PDAC is usually asymptomatic at the early stage, the positive predictive value of CA 19-9 is only 0.9% in this setting [87,88]. Zubarik and colleagues recorded elevated CA 19-9 in 4.9% (n = 27) of subjects in their screening study involving 546 HRIs; neoplastic findings were identified in five individuals on subsequent EUS, and PDAC was diagnosed in one patient (0.2%) [89]. Furthermore, other conditions including benign diseases (pancreatitis, cirrhosis, biliary obstruction, acute cholangitis) and different malignancies (colorectal, gastric, and uterine cancers) can cause increased levels of CA 19-9 [90,91,92,93]. Moreover, CA 19-9 is not synthesized in some people, and only 65% of patients diagnosed with resectable PDAC have increased serum levels [84,94]. Due to the aforementioned, the assessment of CA 19-9 is not recommended for screening, and the CAPS Consortium recommends testing CA 19-9 only in subjects with suspicious findings on imaging and concerns about the presence of PDAC [17]. Nonetheless, its value as a screening tool is being revisited [95].

More recently, novel blood-based biomarkers for early diagnosis have made progress. Studies have confirmed that abnormally expressed serum microRNAs (miRNAs) have certain significance in the diagnosis of early-stage PDAC, or even in precancerous pancreatic lesions [88,96]. The diagnostic value of miRNAs was shown to be higher than that of conventional serum markers [97], and there is evidence that the combination of miRNAs and CA19-9 is more accurate [98,99]. Other emerging methods of detection are so-called “liquid biopsies” that can capture tumor-associated components, such as circulating tumor DNA, extracellular vesicles, and circulating tumor cells. A test utilizing DNA assays and protein biomarkers called CancerSEEK was developed by a team from Johns Hopkins University to detect multiple types of cancer at the same time; regarding PDAC, the test showed a sensitivity of 72% and a specificity of 99% [100]. Another innovative test that combines an eight-plex biomarker signature with CA19-9 is the IMMray PanCan-d assay, which demonstrated an 85% sensitivity and a 98% specificity in distinguishing PDAC stages I and II in HRIs [101]. Its sensitivity and specificity further increased to 89% and 99%, respectively, after excluding Lewis-null individuals from the analysis. These strategies seem very promising, although further studies are needed to verify the results and validity of these methods in clinical practice.

In addition, pancreatogenic (type 3c) DM has recently become a major topic. It refers to DM associated with a disease of the exocrine part of the pancreas. It is most often caused by CP, but it can also be a paraneoplastic manifestation of PDAC [102]. Moreover, it may fit the early diagnosis concept based on the metabolic profile. Sharma and colleagues reported that an increase in fasting blood glucose levels may precede the diagnosis of PDAC by up to 3 years [103]. Furthermore, Sah and colleagues described three distinct phases prior to the diagnosis of PDAC based on metabolic and soft tissue changes: phase one (30–18 months; hyperglycemia) characterized by isolated hyperglycemia, phase two (18–6 months; pre-cachexia) with hyperglycemia and a decrease in serum lipids, body weight, and subcutaneous abdominal fat, and phase three (6–0 months; cachexia) including loss of visceral fat with associated sarcopenia [76]. Abnormal blood glucose values or new-onset DM in an at-risk individual should promptly lead to further diagnostic evaluation [17,104]. Moreover, changes in the blood lipidome signaling the dysregulation of lipid metabolism in pancreatic cancer cells may be determined on mass spectrometry and used particularly to detect PDAC, as it was recently demonstrated by Wolrab and colleagues in their study revealing statistically significant differences between PDAC patients and healthy controls [105]. The sensitivity and specificity to diagnose PDAC were over 90%, which outperformed CA 19-9, especially at the early stage, and were comparable to the established imaging methods.

### 6.3. Goals and Benefits of Pancreatic Screening

The primary goal of pancreatic screening is to reduce mortality associated with PDAC by detecting early stages of the disease and preventing its occurrence by identifying and treating precursor lesions in asymptomatic HRIs [17,23]. In the current practice, PDAC is often metastatic at the time of diagnosis or shortly after the diagnosis is made [4,7]. Published results from established screening programs have shown downstaging of detected PDACs (i.e., more frequent diagnosis of early stages), which was associated with better survival [71,106]. Specifically, Canto and colleagues in their study of 354 HRIs with a median follow-up of 5.6 years identified 14 PDACs, of which 10 (71%) were asymptomatic and 9 of them early and resectable [71]. Four symptomatic patients diagnosed with inoperable PDAC did not undergo examination at the recommended interval. The 3-year survival rate was significantly higher among the nine resectable PDACs compared to the symptomatic group (85% vs. 25%, *p* < 0.0001).

### 6.4. Targets of Pancreatic Screening

The main pathological targets are stage I PDAC (T1–2 N0 M0) and its precursors with high-grade dysplasia, namely pancreatic intraepithelial neoplasia (PanIN) or mucinous cystic lesions (intraductal papillary mucinous neoplasm (IPMN)) [17,23]. Imaging characteristics of IPMN may be useful in identifying dysplastic features, but it is not the case with PanINs, most of which are microscopic lesions that cannot be depicted by conventional methods. It is assumed that most PDACs originate from PanIN lesions [107]. However, PanINs with high-grade dysplasia (formerly PanIN-3) are typically diagnosed only histopathologically, e.g., after surgical resections performed for other pathological findings on imaging. Bartsch and colleagues pointed to the possibility of the presence of highly dysplastic PanIN lesions in patients with multiple small IPMNs elsewhere in the parenchyma [108]; and some small cystic lesions visualized on EUS may be in fact visible PanINs [59,62,63,109]. Of note, the ASGE Standards of Practice Committee has recently suggested that resectable or borderline-resectable PDACs (T1–3 and/or N0–2) were appropriate targets for screening, as the positive impact of screening may be underestimated given that some patients with even locally unresectable cancers may be downstaged with chemoradiation to allow for surgical resection [21].

### 6.5. Risks and Drawbacks of Pancreatic Screening

Potential risks of PDAC screening include adverse events associated with diagnostic procedures, such as EUS-guided fine-needle aspiration (FNA), intravenous application of contrast material, or analgesia/sedation; but those are reported uncommon among the screening study cohorts [21]. Cancer surveillance can lead to patient anxiety, albeit participation in a pancreatic screening program has been shown to reduce cancer anxiety in some [110]. Specifically, Cazacu and colleagues demonstrated positive psychological benefits among HRIs undergoing annual pancreatic screening in their systematic review [111]. Studied individuals reported low-to-moderate cancer-related distress at the beginning that improved substantially over time. Regarding motivation to consider screening, various authors observed that diagnosing an early-stage cancer and contributing to research were the most frequent factors among the subjects [110,112,113]. Lewis and colleagues also discovered that having an affected family relative increased the motivation to participate [112]. Furthermore, their study found that the incentive to go through a particular screening test depended on whether it was recommended by a physician, the degree of invasiveness, its comfort level, and also its cost. Individuals with personal history of other cancers or a positive family history for PDAC often preferred more invasive modalities, expecting these to offer more precise findings. Interestingly, Konings and colleagues reported a slight rise in cancer-worry at a 1-year follow-up that was related to an increased perceived risk of developing cancer and having a relative affected by PDAC before the age of 50 [114].

In addition, potential overdiagnosis or misdiagnosis can occur, resulting in the treatment of completely benign or low-risk neoplastic lesions [25,62,63,93]. The so-called “low-yield surgeries” when histopathology does not reveal pancreatic malignancy or high-grade dysplasia have been considered harmful. Regarding this outcome, the ASGE Standards of Practice Committee identified more than twenty papers from their meta-analysis reporting on rates of low-yield surgeries [21]. The pooled rate of low-yield surgery was estimated to 2.8% (95% CI, 1.9–4.1%; *p* = 0.003) among the whole population of screened individuals; it amounted to 46.6% (95% CI, 34.2–59.4%; *p* = 0.15) in patients who underwent pancreatic surgery as a result of screening (n = 181). These findings were similar to a previous meta-analysis by Paiella and colleagues [115]. However, the question is whether surgical resections of precursor lesions such as low-grade IPMNs should be categorized as harms of screening, given that resection of even low-grade IPMNs may be appropriate to prevent malignant transformation over time in selected young patients with long-life expectancy [21]. 

Disadvantages of the current screening strategies include the dependence on advanced and costly imaging modalities; yet pancreatic screening has been in fact found to be cost-effective for HRIs between 40–76 years of age [116]. Furthermore, “no screening” proved to be the most expensive strategy with minimal benefits according to a recent cost-effectiveness analysis from a Japanese cohort of HRIs [117]. From the point of view of the very essence of pancreatic screening, a significant limitation is the inability to reliably detect and distinguish PanIN lesions by the current methods.

## 7. Management Implications in Identified Lesions

### 7.1. Solid Lesions

Less than 2% of pancreatic lesions identified at baseline screening are solid [59]. In these lesions, pancreatic-protocol CT is indicated [17]. Some indeterminate solid foci detected only on EUS may be PanIN lesions with focally associated lobulo-centric atrophy [109]. Indication for tissue acquisition in solid lesions should be individualized. If the lesion is accessible and the cytological result affects further direction of the patient, it is recommended to perform EUS-FNA [17]; the impact is potentially greater for right-sided lesions of the pancreas requiring pancreaticoduodenectomy.

### 7.2. Cystic Lesions

Approximately one third of HRIs have ≥1 cyst at baseline [59]. Prevalence rises with age, with cystic lesions detected in 14% of individuals less than 50 years old, 34% aged 50–59 years, and 53% aged 60–69 years [59]. Most are low-risk IPMNs and remain unchanged during surveillance [25,68,118]. The approach is generally governed by associated pathological features, and EUS-FNA is indicated in cysts with worrisome features [17].

### 7.3. Changes in MPD Caliber without Visible Lesion

In case an indeterminate MPD stricture without associated mass is detected, CT and alternatively EUS-FNA with repeat imaging within three months is recommended for potential identification of occult neoplasia; ERCP is not recommended [17,63]. However, recent studies pointed out the potential utility of pancreatic juice analysis in the diagnosis of PDAC [77,78], as well as in the prediction of the presence of dysplastic precursor lesions [119,120].

### 7.4. Pancreatic Neuroendocrine Tumors

Screening of HRIs occasionally identifies small (<1 cm) pancreatic neuroendocrine tumors (pNET), although it is not clear whether these lesions are more common in this population than in average-risk individuals [17]. Most incidentally detected pNETs have a low malignant potential [121], and the current guidelines recommend surveillance in asymptomatic non-functioning low-risk pNETs of <2 cm in size [122].

## 8. Surgical Indications

Pancreatic lesions are detected in up to 42% of HRIs [59]. Most of them are managed conservatively and do not require surgical treatment. There was a consensus that indications for pancreatic resection in HRIs should not significantly differ from the established practice in the general population, and decision-making should be made within multidisciplinary teams [17]. It should be borne in mind that abnormalities with zero to low malignant potential are detected far more often than clinically relevant lesions.

All pancreatic lesions suspected of being PDAC should be resected [17]. However, consensus on the surgical approach in HRIs undergoing resection for a suspected PDAC is evolving. Most experts do not recommend total pancreatectomy unless it is necessary to achieve a completely negative (R0) resection margin [17]. The occurrence of PanIN within surgical margins is a subject of discussion. The data suggest that PanIN of any degree at the margins of a resected pancreas with invasive PDAC has no prognostic consequences; however, the clinical significance of dysplasia at the margins of a resected pancreas without invasive PDAC needs to be determined [123,124]. Some experts would not perform further surgery [123], but in the case of PanIN with high-grade dysplasia, follow-up imaging is recommended within 6 months of surgery and then regularly “indefinitely” due to the risk of new or metachronous neoplasias in these patients [17]. Canto and colleagues published results of HRI patients post-resection of screening-detected neoplasms [125]. Out of the total of 354 subjects, 48 were operated on (22 solid lesions, 25 cysts, 1 MPD stricture). Most underwent partial pancreatectomy; metachronous PDAC developed in the remnant parenchyma of two patients, both of whom had prior surgery for benign precursor lesions. The median length of hospital stay was 7 days, the rate of postoperative adverse events was 35%, and the perioperative mortality was zero, which was comparable to that for standard indications. Importantly, nine out of ten detected PDACs were resectable, with a 5-year survival rate of 60%.

Surgical resection is recommended for the following [17]:

- Solid pancreatic lesion ≥ 5 mm of indeterminate pathology
- Positive or highly suspicious FNA result (except for non-functioning pNET)
- Cystic lesion with worrisome features suspicious of malignancy (mural nodules, an enhancing solid component, MPD dilatation of ≥ 10 mm, an abrupt MPD caliber change with distal atrophy, associated symptoms of pancreatitis, jaundice, or pancreatic pain)

### Surveillance of Individuals without Indication for Surgery

The recommended management algorithm is outlined in Table 2 [15,17,19,21,23]. Individuals without concerning abnormalities should undergo follow-up imaging in 12 months and screening should continue as long as they are surgical candidates [15,17,19,21,23]. Surveillance intervals for concerning pathologies that do not show signs of malignancy (provided that CT or FNA were performed) are determined by the size, number, and type of lesions, their growth rate, and related features [15,17,18]. Repeat imaging in 3 months should be performed if any of the following are present: (a) solid lesion < 5 mm of uncertain significance, (b) solid lesion with MPD width of 5–9 mm, and (c) MPD stricture and/or dilatation ≥ 6 mm of unclear etiology. Repeat imaging in 6 months is recommended in the case of: (a) cystic lesion ≥ 3 cm in size, (b) cystic lesion with MPD width of 5–9 mm, (c) cystic lesion with associated lymphadenopathy, (d) cyst growth rate ≥ 5 mm/2 years, and (e) elevated serum CA 19-9 level.

## 9. Conclusions

Pancreatic cancer remains one of the deadliest malignancies with dismal prognosis and limited options for effective therapy. The grim reality is that most patients with PDAC have advanced or metastatic disease at diagnosis. Early detection is thus considered to be the most effective way to improve survival, as radical surgery is the only potential curative procedure. Population-based screening is currently not recommended, however, identification of HRIs defined by genetic and familial risk and utilization of minimally invasive screening modalities, namely annual EUS or MR/MRCP, is justified.

The role of blood-based tests needs to be further studied. Future goals should be focused on finding molecular markers that reliably suggest the presence of incipient (premalignant) lesions even if the neoplasms are too small to be depicted on imaging (i.e., PanINs). Furthermore, it is crucial to aggregate all present risk factors in each individual and perhaps change the paradigm with greater emphasis on prevention.

Lastly, more data are needed regarding the natural history of precursor lesions in HRIs and the impact of screening programs on morbidity and mortality in this population. For these reasons, it is recommended to implement screening programs in the setting of research protocols. The success of such programs requires patient compliance as well as multidisciplinary cooperation of expert endosonographers, pancreatobiliary surgeons, dedicated gastroenterologists, radiologists, histopathologists, oncologists, and clinical geneticists.

## Figures and Tables

**Table 1:** Screening recommendations by expert societies for individuals with an inherited risk of pancreatic cancer [17,19,21].

	CAPS	ACG	ASGE
Peutz–Jeghers syndrome	Regardless of family history; start at age 40 years (or 10 years younger than earliest PDAC in family).	Regardless of family history; start at age 35 years (or 10 years younger than earliest PDAC in family).	Family history criteria n/a; start at age 35 years (or 10 years younger than earliest PDAC in family).
FAMMM syndrome	Regardless of family history; start at age 40 years (or 10 years younger than earliest PDAC in family).	Regardless of family history; start at age 50 years (or 10 years younger than earliest PDAC in family).	Family history criteria n/a; start at age 40 years (or 10 years younger than earliest PDAC in family).
HBOC syndrome	≥1 FDR (BRCA1 *, *BRCA2*, *PALB2*) or ≥2 relatives † of any degree (*BRCA2*) with PDAC; start at age 45–50 years (or 10 years younger than earliest PDAC in family).	First- or second-degree relative with PDAC; start at age 50 years (or 10 years younger than earliest PDAC in family).	Regardless of family history (*BRCA1*, *BRCA2*), not specified for *PALB2*; start at age 50 years.
Lynch syndrome	≥1 FDR with PDAC; start at age 45–50 years (or 10 years younger than earliest PDAC in family).	First- or second-degree relative with PDAC; start at age 50 years (or 10 years younger than earliest PDAC in family).	First- or second-degree relative with PDAC; start at age 50 years (or 10 years younger than earliest PDAC in family).
Ataxia telangiectasia	≥1 FDR with PDAC; start at age 45–50 years (or 10 years younger than earliest PDAC in family).	First- or second-degree relative with PDAC; start at age 50 years (or 10 years younger than earliest PDAC in family).	First- or second-degree relative with PDAC; start at age 50 years (or 10 years younger than earliest PDAC in family).
Hereditary pancreatitis	Did not reach consensus but stated that most experts recommended screening at age 40 years or 20 years after the first pancreatitis attack; regardless of gene status.	Start at age 50 years (or 10 years younger than earliest PDAC in family).	Start at age 40 years (with CT or MR, as early tumors may be obscured by fibrosis and calcifications on EUS); autosomal dominant variants.
Familial pancreatic cancer	≥2 relatives with PDAC of whom ≥1 is FDR; start at age 50–55 years (or 10 years younger than earliest PDAC in family).	≥2 relatives with PDAC of whom ≥1 is FDR or ≥3 relatives with PDAC; start at age 50 years (or 10 years younger than earliest PDAC in family).	FPC kindreds; start at age 50 years (or 10 years younger than earliest PDAC in family, whichever comes first).

CAPS—International Cancer of the Pancreas Screening Consortium, ACG—American College of Gastroenterology, ASGE—American Society for Gastrointestinal Endoscopy, FAMMM—familial atypical multiple mole and melanoma, HBOC—hereditary breast and ovarian cancer, PDAC—pancreatic ductal adenocarcinoma, FDR—first-degree relative, CT—computed tomography, MR—magnetic resonance, EUS—endoscopic ultrasonography, FPC—familial pancreatic cancer; * grade 3 recommendation, 69.6% agreement; † wherever a relative is stated, this indicates blood relatives only.

**Table 2:** Recommended algorithm of pancreatic cancer screening in high-risk individuals [15,17,19,21,23].

**At baseline**	
• EUS + MR/MRCP	• Fasting blood glucose and/or HbA1c
**During follow-up**	
• EUS + MR/MRCP (*)	• Fasting blood glucose and/or HbA1c
**If indicated**	
• Serum CA 19-9	Concerning features on imaging
• EUS-FNA	Solid lesion ≥ 5 mm
	Cystic lesion with worrisome features
	Unclear MPD stricture and/or dilatation ≥ 6 mm
• CT scan	Solid lesions (regardless of size)
	Unclear MPD stricture and/or dilatation ≥ 6 mm
**Surveillance intervals**	
• 12 months	No concerning abnormalities (e.g., cysts without worrisome features)
• 3–6 months	Concerning pathologies without signs of malignancy (see text)
**Surgical resection**	
• Positive FNA or high suspicion of malignancy on imaging (see text)	

EUS—endoscopic ultrasonography, MR/MRCP—magnetic resonance with magnetic resonance cholangiopancreatography, HbA1c—hemoglobin A1c, FNA—fine-needle aspiration, MPD—main pancreatic duct, CT—computed tomography. * There is no consensus on if and how to alternate EUS and MR/MRCP.

## Data Availability

Not applicable.
